# Refined Matrix Completion for Spectrum Estimation of Heart Rate Variability

**DOI:** 10.3934/mbe.2024296

**Published:** 2024-08-02

**Authors:** Lei Lu, Tingting Zhu, Ying Tan, Jiandong Zhou, Jenny Yang, Lei Clifton, Yuan-Ting Zhang, David A. Clifton

**Affiliations:** 1 School of Life Course & Population Sciences, King’s College London, London, UK, WC2R 2LS; 2 Department of Engineering Science, University of Oxford, Oxford, UK, OX1 2JD; 3 Department of Mechanical Engineering, The University of Melbourne, Parkville, VIC, Australia, 3010; 4 Department of Family Medicine and Primary Care, Li Ka Shing Faculty of Medicine, The University of Hong Kong, Hong Kong, China; 5 School of Public Health, Li Ka Shing Faculty of Medicine, The University of Hong Kong, Hong Kong SAR, China; 6 Department of Pharmacology and Pharmacy, The University of Hong Kong, Hong Kong SAR, China; 7 Nuffield Department of Clinical Medicine, Experimental Medicine Division, University of Oxford, Oxford, UK; 8 Department of Electronic Engineering, Chinese University of Hong Kong, Hong Kong SAR, China; 9 Oxford Suzhou Centre for Advanced Research, Suzhou, China

**Keywords:** Heart rate variability, spectrum estimation, matrix completion, uncertainty, HRV modelling

## Abstract

Heart Rate Variability (HRV) is an important metric in cardiovascular health monitoring. Spectral analysis of HRV provides essential insights into the functioning of cardiac autonomic nervous system. However, data artefacts could degrade signal quality, potentially leading to unreliable assessments of cardiac activities. In this study, we introduce a novel approach for estimating uncertainties in HRV spectrum based on matrix completion. The proposed method utilises the low-rank characteristic of the HRV spectrum matrix to efficiently estimate its uncertainties. In addition, we developed a refined matrix completion technique to enhance the estimation accuracy and computational cost. Benchmarking on five public datasets, our model shows effectiveness and reliability in estimating uncertainties in HRV spectrum, and has superior performance against five deep learning models. The results underline the potential of our developed method in providing reliable HRV spectrum uncertainty estimation.

## 1 Introduction

Heart Rate Variability (HRV) is the measure of fluctuations between successive heartbeats, reflecting the interplay between the sympathetic and parasympathetic branches of the autonomic nervous system (ANS) [1]. These fluctuations are critical markers of the physiological factors influencing heart rhythm, serving as indicators of an individual’s health status [2]. Studies have shown that higher HRV levels are indicative of robust health, suggesting a well-balanced autonomic regulation. In contrast, reduced HRV is associated with an imbalance in autonomic function, leaning towards either diminished parasympathetic activity or heightened sympathetic drive, which correlates with an increased risk of adverse health outcomes [3, 4]. The relevance of HRV extends to its application in diagnosing and monitoring cardiovascular conditions, which are leading contributors to global mortality [5]. This underscores the importance of HRV as an important parameter in the evaluation of both physiological well-being and the presence of pathological conditions, particularly those related to cardiovascular health.

HRV data analysis can be implemented through various signal processing techniques. In the time domain, HRV is assessed by calculating statistical measures such as the average of normal-to-normal (NN) intervals, the root mean square of successive differences between NN intervals (RMSSD), and the standard deviation of NN intervals (SDNN) [6]. These metrics provide insights into the heart rhythm and its variability over time. On the other hand, frequency domain analysis of HRV offers a perspective on the distribution of power across different frequency bands within the HRV data, serving as a more detailed reflection of autonomic nervous system activity. Spectral analysis has proven effective in distinguishing between sympathetic and parasympathetic nervous system influences on heart rate variability [7]. Specifically, the low-frequency (LF) component of the HRV spectrum is influenced by both sympathetic and parasympathetic activities, whereas the high-frequency (HF) component is predominantly a result of parasympathetic activity. Consequently, the LF/HF ratio is commonly utilised as a measure of the balance between sympathetic and parasympathetic influences, offering valuable insights into autonomic nervous system dynamics [8].

The most commonly used method to derive HRV data is via electrocardiogram (ECG) signals. With appropriate QRS detectors, R-peaks in the ECG morphology can be identified, then the HRV data can be obtained by computing the RR intervals [9]. Other than using ECGs, some studies suggest using photoplethysmography (PPG) signals to derive the HRV data, where PPG is a non-invasive technique that utilises optical principles to obtain pulse waves from the microcirculation in peripheral tissue [10]. In particular, the utilisation of PPG signals for detecting pulse-wave related HRV, known as pulse rate variability (PRV), has attracted considerable attention in recent years [11]. This is primarily attributed to the convenience and affordability of acquiring PPG signals through wearable devices.

While Electrocardiogram (ECG) or Photoplethysmography (PPG) signals are commonly employed for HRV analysis, ensuring the reliability of HRV data extracted from these signals presents significant challenges. These difficulties are largely due to uncertainties inherent in data collection and computational methodologies, which may compromise data integrity. In particular, motion artefacts represent a substantial challenge, often overlapping in frequency with legitimate HRV signals (0.5 to 5 Hz) as they typically manifest within a 0.01 to 10 Hz range [12]. This overlap complicates the task of artefact removal without inadvertently altering the PPG signal. Moreover, the processes involved in HRV data analysis, such as the identification of R-peaks, interpolation of RR intervals, and data resampling, introduce additional complexity. These steps are crucial for accurate HRV measurement but can potentially distort the HRV data, highlighting the intricate balance required in processing and analysing HRV signals.

A variety of computational methods have been devised to mitigate uncertainties or inaccuracies in HRV data analysis, including adaptive filtering, blind source separation, and advanced deep learning techniques [13, 14, 15]. Despite the sophistication of these approaches, they often overlook the unique properties of the HRV spectrum’s frequency bands, which limits their effectiveness in accurately estimating uncertainties. For instance, the low-frequency (LF) component of the HRV spectrum is influenced by both sympathetic and parasympathetic nervous system activities, whereas the high-frequency (HF) component is primarily regulated by the parasympathetic nervous system (PNS) [8]. Research has shown that measurement noise impacts these frequency bands; For example, the LF component exhibits substantial sensitivity to increasing noise levels, while the impact is not obvious in the HF component of the HRV spectrum [16]. This differential impact highlights the necessity of accounting for the distinct characteristics of HRV frequency bands in the development and application of computational techniques for HRV data analysis.

In this study, we introduce a novel approach for estimating uncertainties in the HRV spectrum by leveraging a refined low-rank matrix completion (MC) technique, which incorporates the distinct characteristics of HRV frequency components to improve the precision of uncertainty estimation. The concept of low-rank MC emerges as a powerful strategy for reconstructing missing or imprecise entries in observed data, grounded in the principle that many real-world datasets exhibit a low-rank structure due to their inherent low-dimensional characteristics or underlying patterns [17, 18]. The application of low-rank properties to data is widely recognised in various domains, indicating the existence of underlying trends or dimensions within extensive datasets [19]. The MC method has demonstrated its efficacy in various fields, including enhancing image quality in compressive sampling photoacoustic microscopy [20], optimising signal channel selection in electroencephalogram analysis [21], and facilitating the identification of protein-protein interactions [22]. While there have been initial explorations of MC techniques in analysing HRV data [23, 24], its potential in healthcare remains largely untapped, with a significant gap in detailed model development and comprehensive validation of its performance in medical applications.

By leveraging the low-rank characteristic of HRV data, we demonstrated the effective inference of uncertainties within the HRV spectrum using partial entries from the data matrix. This approach is further enhanced by a detailed analysis of HRV data characteristics, particularly focusing on the low-frequency (LF) and high-frequency (HF) components of the spectrum. Drawing on these insights, we developed a refined matrix completion (RMC) framework specifically designed to improve the estimation of uncertainties. This framework prioritises critical data entries within the HRV matrix, thereby enhancing the method’s efficiency and robustness in estimating uncertainties. To assess the efficacy of our proposed RMC method in HRV spectrum estimation, we conducted comprehensive experimental studies across five benchmark datasets. Our method was evaluated against both the traditional MC approach and five regression-based machine learning models. The findings from these experiments and comparative analyses underscore the superior performance and robustness of our RMC method in HRV spectrum estimation. The key contributions of this study include:

- The introduction of an innovative method for estimating uncertainties in the HRV spectrum, utilising the matrix completion technique to exploit the low-rank nature of the HRV data.
- The development of an advanced matrix completion framework that enhances estimation accuracy and computational efficiency by focusing on the modelled spectrum of HRV data.
- The implementation of extensive experimental validation on five renowned benchmark datasets to evaluate the effectiveness of our model in HRV spectrum estimation.
- A series of comparative analyses with various neural network models and the traditional MC method, illustrating the significant advantages of our approach in the context of uncertainty estimation.

The remainder of this paper is organised as follows. Section 2 formulates the problem of HRV spectrum estimation. Section 3 develops the MC method and refinement. Section 4 performs the analysis of HRV data. The results of spectrum estimation and comparison study are presented in Section 5, and finally conclusions are drawn in Section 6.

## 2 Problem Formulation

### 2.1 Notations

In this paper, we denote the set of real numbers by ℝ and the set of natural numbers by 𝒩. A matrix is represented by a bold uppercase letter **A**, which consists of elements *a*_*i, j*_ for *i* = 1, …, *n* and *j* = 1, …, *p*, and belongs to the space ℝ^*n*×*p*^, indicating it has *n* rows and *p* columns. The Frobenius norm of matrix **A** is expressed as $||A{||}_{F}={\left(\sum_{i=1}^{n}\sum_{j=1}^{p}{\left|a_{ij}\right|}^{2}\right)}^{1/2}$, encapsulating the square root of the sum of the absolute squares of its elements. The nuclear norm of matrix **A** is defined as ||**A**||* = ∑_*i*_ *σ_i_*(**A**), where *σ_i_*(**A**) represents the *i_th_* singular value of **A**. The 2-norm of **A** is denoted as ||**A**||_2_ = *σ*_max_(**A**), with *σ*_max_ being its largest singular value.

### 2.2 Problem Formulation

Assuming that *z*(*t*) ∈ ℝ, for *t* [0, *T*_*s*_], represents the pre-processed HRV data over a time span of *T*_*s*_, its frequency components can be delineated as follows:

(2.1) $$P(f,\tilde{f})=ℱ(z(t)),$$

where $\tilde{f}=\left[f_{l},f_{r}\right]$ specifies the frequency range of interest within the HRV data, with *f*_*l*_ and *f_r_* denoting the left and right boundaries, respectively. Here, ℱ(·) symbolises the spectrum operation tailored to the selected frequency range $\tilde{f}$, and $P(f,\tilde{f})$ represents the power spectral density (PSD) function of the HRV data, where various frequency-related HRV metrics can be extracted. More precisely,

(2.2) $$h_{k}\left(f,{\tilde{f}}_{k}\right)=\phi_{k}\left({\tilde{f}}_{k},P(f,\tilde{f})\right),$$

where *k* = 1, 2, …, *N_k_* indicates different types of HRV variables, such as the power of LF or HF, or the ratio between the LF and HF. The nonlinear mapping *ϕ_k_*(·, ·) computes frequency variable *h_k_* from the spectrum *P*(·, ·) for the *k^th^* type of HRV variables in the ${\tilde{f}}_{k}$ frequency range.

It is important to note that the accuracy of HRV measurements is influenced by various uncertainties, including noise and missing values. Additional factors, such as respiration rate, circadian rhythms, and physiological states, can also affect the data quality, therefore impacting the frequency analysis of HRV data. Consequently, these uncertainties pose challenges in precisely estimating the HRV spectrum.

Given data collected from *N*_*s*_ subjects, we construct a matrix $H={\left\{h_{i,k}\right\}}_{i=1,\ldots ,N_{s},k=1,\ldots ,N_{k}}\in {\mathbb{R}}^{N_{s}\times N_{k}}$, derived from the measurements, where each element is defined by

(2.3) $$h_{i,k}\left(f,{\tilde{f}}_{k}\right)=\phi_{k}\left({\tilde{f}}_{k},P_{i}(f,\tilde{f})\right),$$

where *P_i_* is the PSD of the *i_th_* subject.

The PSD often reveals predominant frequency components, with many of the non-dominant frequencies having samll amplitudes that are considered negligible, resulting in a sparse matrix. In addition, the HRV spectrum matrix is derived from the time series of intervals between heartbeats. As the ECG waveform has similar repeating patterns [25], the different spectral components derived from ECG data would not be completely independent. This leads to the rank of the matrix is lower than the dimensions of the matrix, suggesting the low-rank characteristic of the HRV spectrum matrix. This observation underpins our motivation to apply low-rank MC for approximating the matrix and estimating uncertainties. This motivates us to introduce the RMC approach aiming at more effectively estimation of the uncertainties within the spectrum. This step represents an advancement in our methodology, leveraging the foundational concept of low-rank MC while incorporating adjustments to enhance the precision and efficiency of uncertainty estimation in HRV spectrum analysis.

## 3 Methodology

This section delves into the application of low-rank MC technique for handling sparse matrices. The core principle revolves around identifying an effective approximation for the matrix that aligns with certain predefined cost functions. One common approach involves seeking an optimal approximation that minimises the matrix rank. The section starts with a revisit of low-rank MC, followed by the matrix approximation with the interested zone, and then the introduction of the RMC approach.

### 3.1 Low-rank Matrix Completion

Assuming matrix $H\in {\mathbb{R}}^{N_{s}\times N_{k}}$ is sparse, i.e., only a limited number of entries in the matrix are non-zero. We denote the set Ω as the collection of non-zero entries of the matrix **H**, which is represented as

(3.1) $$\text{Ω}=\left\{(i,k)|h_{i,k}\neq 0,i=1,\ldots N_{s},k=1,\ldots ,N_{k}\right\}.$$

The spectra **H**, derived from segmenting HRV data sequences, often contain redundant information due to the recursive slicing approach used to generate these segments. This redundancy suggests that not all information within the matrix is equally valuable for analysis. To address this, we employ a low-rank matrix completion technique, which is particularly adept at preserving essential information while minimizing redundancy [17, 26]. It can be formulated by approximating matrix **H** in terms of the smallest rank, i.e.,

(3.2) $$\begin{array}{l} \begin{array}{cc} \min & \text{rank}(X) \end{array}\\ \begin{array}{ccc} s.t. & x_{i,k}=h_{i,k}, & \text{for}(i,k)\in \Omega , \end{array} \end{array}$$

where matrix $X={\left\{x_{i,k}\right\}}_{i=1,\ldots ,N_{s},k=1,\ldots ,N_{k}}\in {\mathbb{R}}^{N_{s}\times N_{k}}$ is an optimal approximation matrix that can keep the majority information of **H**.

The optimisation of rank-minimization problem in Eq. (3.2) is generally computationally intractable. Instead of solving the original problem of the rank norm, a convex relaxation based on the nuclear norm is given by [17],

(3.3) $$\begin{array}{l} \begin{array}{cc} \min & ||X{||}_{*} \end{array}\\ \begin{array}{ccc} s.t. & x_{i,k}=h_{i,k}, & \text{for}(i,k)\in \text{Ω}, \end{array} \end{array}$$

where, || · ||* indicates the nuclear norm of matrix **X**, which is defined as the summation of its singular values. In particular, it is demonstrated that a smaller nuclear norm indicates the lower rank of the matrix [17]. However, it is generally too harsh to force the estimation of all observed entries in the matrix, so alternatively, a more practical approximation of Eq. (3.3) can be formulated by relaxing the constraint [27, 28].

(3.4) $$\begin{array}{l} \begin{array}{cc} \min & ||X{||}_{*} \end{array}\\ \begin{array}{cc} s.t. & ||{𝒜}_{\text{Ω},H}(X)-{𝒜}_{\text{Ω},H}(H)|{|}_{F}\leq \delta , \end{array} \end{array}$$

where || · ||_*F*_ denotes the Frobenius norm, *δ* is a small value to constrain the estimation, and 𝒜_Ω,**H**_(·) is a projection operator that is defined as

(3.5) $${𝒜}_{\text{Ω},H}(X)=\{\begin{array}{cc} x_{i,k}=h_{i,k} & (i,k)\in \text{Ω}\\ 0 & \text{otherwise}. \end{array}$$

It is noted that Eq. (3.4) can be formulated as a penalised least-squares convex program [29], which can be solved by the singular value thresholding (SVT) technique as described in [30].

### 3.2 Matrix Approximation with Interested Zone

The method previously outlined operates under the assumption that the observed entries, including those missing, are random variables that follow a uniform distribution [17]. This assumption does not fully account for the effects of measurement uncertainties on different frequency components of the data, which often deviate from a uniform distribution pattern [16]. Therefore, the traditional lowrank MC techniques mentioned above could not be applied directly. Alternatively, the interest-zone matrix-approximation (IZMA) method [26] is proposed to solve the low-rank MC problem when some columns/sub-columns or rows/sub-rows are missing. More specifically, the IZMA tries to solve the following optimization problem,

(3.6) $$\begin{array}{cc} \min & ∥{𝒜}_{\text{Ω},H}(X)-{𝒜}_{\text{Ω},H}(H)|{|}_{F}\\ s.t. & p(X)\leq 0 \end{array}$$

where the nonlinear mapping $p(\cdot ):{\mathbb{R}}^{N_{s}\times N_{k}}\to {\mathbb{R}}^{n}$ is a smooth function for some *n* ∈ 𝒩. For example, *p*(**X**) can be some constraints with respect to the estimated matrix, such as *p*(**X**) = ||**X**||_2_ − *λ, λ* > 0. Another example of this nonlinear mapping is *p*(**X**) = ||**X**|| * − *λ* for some *λ* > 0 as described in [26]. The optimisation problem in Eq. (3.6) can be solved by the following steps.

It first approximates the following optimisation for the constrained full matrix,

(3.7) $$\begin{array}{cc} \min & ||X-H{||}_{F}\\ s.t. & p(X)\leq 0, \end{array}$$

The solution to the optimisation in Eq. (3.7) with the spectral norm constraint can be estimated by,

(3.8) $$\{\begin{array}{l} \tilde{X}=U\tilde{\Sigma }V^{*},\\ \tilde{\Sigma }=\text{diag}\left({\tilde{\sigma }}_{i}\right), \end{array}$$

where matrices **U** and **V** are calculated by **H** = **U**ΣV*, with Σ = diag(*σ*_1_, *σ*_2_, …, *σ*_*n*_). Next, the diagonal matrix is obtained as ${\tilde{\sigma }}_{i}=\min\left(\sigma_{i},\lambda \right)$, *i* = 1, 2,, …, *k*, and the approximation $\tilde{X}$ can be obtained by the data reconstruction.

Then, the estimation of matrix **H** with entries in the interested zone can be updated as follows,

(3.9) $$ℋ(X):=ℐ(\tilde{X})-{𝒜}_{\text{Ω},H}(\tilde{X})+{𝒜}_{\text{Ω},H}(H),$$

where ℐ is an identity operator, and ℋ(·) denotes the updating process, which combines the known entries in matrix **H** and the estimated entries in matrix $\tilde{X}$.

Next, the estimated matrix is replaced with ℋ(**X**) and the iteration is repeated starting from Eq. (3.7). The estimation is evaluated by $\epsilon (X)≜||{𝒜}_{\text{Ω},H}(X)-{𝒜}_{\text{Ω},H}(H)|{|}_{F}$, and missing entries in matrix **H** can be approximated until *ϵ*(**X**) converges. For convenience, we denote the whole iteration process of the constrained approximation as 𝒯 (**H**, *λ*), which will be used in the next section for the HRV spectrum estimation.

It is noted that the matrix approximation in Eq. (3.6) is different from the low-rank MC as presented in Eq. (3.2), where the low-rank MC requires to fix the known entries for the estimation; while the matrix approximation in Eq. (3.6) minimise the objective function with Frobenius norm to the constraint function. In particular, the technique of matrix approximation with an interested zone can also be extended as matrix completion, which develops a new iteration process separate from the traditional SVT technique; more details of the iteration process can be found in [26], and it will also be discussed in Section 3.3.

The process of approximating missing entries in this context is driven by the data itself through an iterative approach. It is important to acknowledge that in HRV data analysis, certain mathematical models can be leveraged to represent the spectrum effectively. One such model, the Gaussian model [31], has been highlighted for its capability to accurately characterise HRV spectra and elucidate the relationships between various spectrum components. In the subsequent subsection, we will apply the Gaussian model to refine the RMC process by constructing a new matrix that possesses significantly reduced dimensions. This approach is anticipated to not only enhance the efficiency of the estimation process, but also to improve its overall performance by providing a more focused and computationally manageable framework for addressing missing data within the HRV spectrum analysis.

### 3.3 Model-based Refined MC for HRV Spectrum Estimation

**Figure 1:**
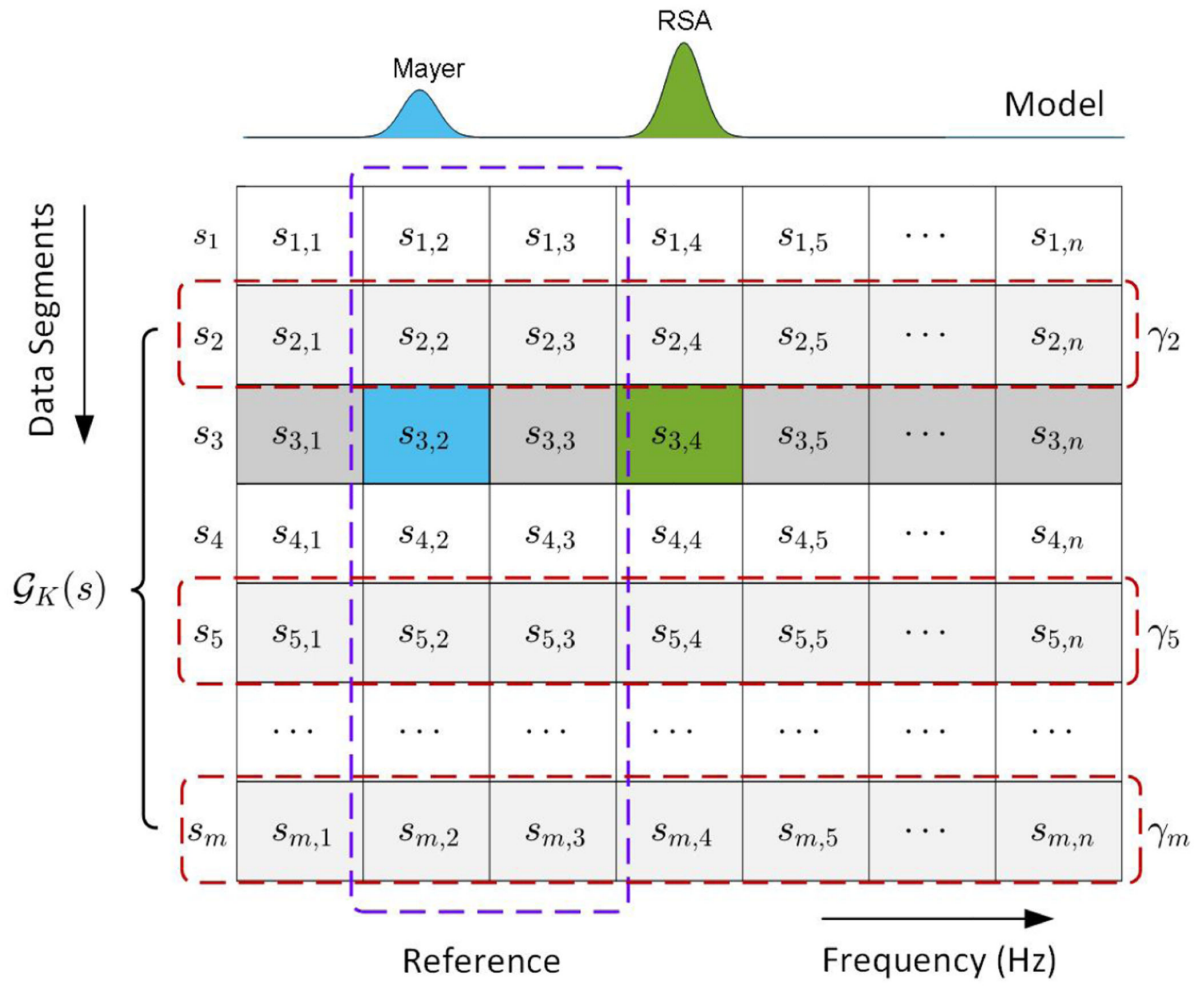
Illustration of MC and RMC using model information for HRV spectrum estimation.

The HRV data spectrum is characterised by distinct frequency bands, each with unique physiological implications. As illustrated in Figure 1, the HF band captures the influence of respiration on heart rate, a phenomenon known as respiratory sinus arrhythmia (RSA); Conversely, the LF band encompasses oscillations associated with blood pressure regulation and vasomotor tone, often referred to as Mayer waves. These distinctions provide a foundation for modelling the HRV spectrum, allowing for a nuanced understanding of the underlying physiological processes. With these considerations, we model the spectrum of HRV data as follows.

We assume the matrix $S={\left\{s_{i,\ell }\right\}}_{i=1,\ldots ,n_{r},\ell =1,\ldots ,n_{fr}}\in {\mathbb{R}}^{n_{r}\times n_{fr}}$ consists of *n_r_* spectra that are derived from the HRV data, where $s_{i}={\left[\begin{array}{ccc} s_{i,1}, & \cdots , & s_{i,n_{f_{r}}} \end{array}\right]}^{T}\in {\mathbb{R}}^{n_{f_{r}}}$ is a vector of the spectrum. In particularly, for each **s**_*i*_ ∈ **S**, it can be modelled with Gaussian functions [31], and the entry *s*_*i,𝓁*_ can be represented as,

(3.10) $$s_{i,\ell }^{m}\left(f,A_{i,\ell },\sigma_{i,\ell },f_{i,\ell }\right)=\frac{A_{i,\ell }}{\sqrt{2\pi }\sigma_{i,\ell }}\exp^{\left(-\frac{{\left(f-f_{i,\ell }\right)}^{2}}{2\sigma_{i,\ell }^{2}}\right)},$$

where *f* ∈ [*f*_*l,i*_, *f*_*r,i*_] indicates the interval of the frequency band of interests, i.e., LF or HF spectrum. Consequently, *A*_*i,𝓁*_ > 0 is the weight of amplitude, *f*_*i,𝓁*_ > 0 is the peak position of frequencies in the spectrum, and *σ*_*i,𝓁*_ > 0 is its standard deviation for this frequency. For simplicity of notations, $s_{i,\ell }^{m}$ is used to represent $s_{i,\ell }^{m}\left(f,A_{i,\ell },\sigma_{i,\ell },f_{i,\ell }\right)$ when no confusion arises, and ${\tilde{s}}_{i}^{m}={\left\{s_{i,\ell }^{m}\right\}}_{\ell =1,\ldots ,n_{fr}}$ indicates the modeled spectrum.

It is noted that the HRV spectrum can be characterised with different frequency components, and they can be simulated by changing the frequency intervals of the modelling, after which the complete spectrum can be obtained by combining these different frequency components. Next, we use the modelled information to define a new matrix for the estimation of missing entries. For the convenience of presentation, it is assumed that some entries of matrix **S** are missing, and the set ${𝒮}_{m}={\left\{s_{j}\right\}}_{j=1,\ldots ,n_{q}}$ contains the rows in **S** with missing values.

For any **s**_*j*_ ∈ *𝒮*_*m*_, we model the spectrum and obtain ${\tilde{s}}_{j}^{m}$ with Eq. (3.10), and then calculate the correlation between ${\tilde{s}}_{j}^{m}$ and ${\tilde{s}}_{q}^{m}$ spectra, where ${\tilde{s}}_{q}^{m}$ is the modelled information of spectrum in **S**, and the correlation is computed as follows,

(3.11) $$\gamma \left(s_{j,\ell_{t}}^{m},s_{q,\ell_{t}}^{m}\right)=\frac{\sum_{\ell_{t}=1}^{N_{1}}\left(s_{j,\ell_{t}}^{m}-{\bar{s}}_{j}^{m}\right)\left(s_{q,\ell_{t}}^{m}-{\bar{s}}_{q}^{m}\right)}{\sqrt{\sum_{\ell_{t}=1}^{N_{1}}{\left(s_{j,\ell_{t}}^{m}-{\bar{s}}_{j}^{m}\right)}^{2}}\sqrt{\sum_{\ell_{t}=1}^{N_{1}}{\left(s_{q,\ell_{t}}^{m}-{\bar{s}}_{q}^{m}\right)}^{2}}},$$

where ${\bar{s}}_{j}^{m}=\frac{1}{N_{1}}\sum_{\ell_{t}=1}^{N_{1}}s_{j,\ell_{t}}^{m}$ and ${\bar{s}}_{q}^{m}=\frac{1}{N_{1}}\sum_{\ell_{t}=1}^{N_{1}}s_{q,\ell_{t}}^{m}$ are means of the two spectra respectively, *𝓁*_*t*_ = 1, …, *N*_1_, $N_{1}\leq n_{f_{r}}$ is the index of known components of the spectrum, and $\gamma_{j,q}=\gamma \left(s_{j,\ell_{t}}^{m},s_{q,\ell_{t}}^{m}\right)$ indicates the relationship between the two spectra.

After calculating *γ*_*j,q*_ for the **s**_*j*_ spectrum, a new vector $t_{j}=\left[\begin{array}{c} \gamma_{j,1}\cdots \gamma_{j,n_{r}-1} \end{array}\right]$ can be obtained, we sort **t**_*j*_ and select the *K* highest ranked elements for **s**_*j*_, then a set that contains the identified indices can be formed as,

(3.12) $${𝒢}_{K}\left(s_{j}\right)=\left\{g_{k}|t_{j,g_{k}}\in t_{j},g_{k}\in 𝒥,k=1,\ldots ,K\right\},$$

where 𝒥 = 1, …, *n_r_* − 1 denotes the index of spectrum in matrix **S**.

Next, a refined matrix $S_{{𝒢}_{K}}\in {\mathbb{R}}^{K\times n_{f_{r}}}$ can be formulated by combining **s**_*j*_ and the identified HRV spectra. Consequently, the optimisation in Eq. (3.6) can be updated as,

(3.13) $$\begin{array}{l} \min{𝒜}_{\tilde{\Omega },S_{G_{K}}}∥(X_{{𝒢}_{K}})-{𝒜}_{\tilde{\Omega },S_{G_{K}}}(S_{{𝒢}_{K}}){∥}_{F}\\ \text{s}.t.p(X_{{𝒢}_{K}})\leq 0, \end{array}$$

where $\tilde{\Omega }$ is the set of observed entries in the refined matrix, ${𝒜}_{\tilde{\Omega },S_{{𝒢}_{K}}}(\cdot )$ is the updated operator to model the missing values, $X_{{𝒢}_{K}}$ indicates the estimated matrix, and the spectral norm $p\left(X_{{𝒢}_{K}}\right)=X_{{𝒢}_{K}}2-\lambda$ can be used for the constraint.

For the estimation of missing entries in the refined matrix, we first approximate the following constrained full matrix,

(3.14) $$\begin{array}{c} \min||X_{{𝒢}_{K}}-S_{{𝒢}_{K}}|{|}_{F}\\ s.t.p\left(X_{{𝒢}_{K}}\right)\leq 0, \end{array}$$

where the soft imputing method as described in Eq. (3.8) can be used to solve the estimation in Eq. (3.14). Then, by updating the parameter *λ* in the constraint function, missing entries in the refined matrix can be estimated using the iteration process as described in Section 3.2. Algorithm 1 presents the pseudo code of the proposed RMC method. The hyperparameters tolerance *e*_*tol*_ and *λ*_*tol*_ are set as 10^-8^ and 10 according to the configuration in [26].

As an illustration, Figure 1 demonstrates the concept of estimating HRV spectrum using the RMC technique, where the matrix is constructed with *m* spectra that are derived from HRV data segments. The *x*-axis of the matrix indicates the frequency band of interests, and *y*-axis shows the indices of data segments. Suppose the HF band of **s**_3_ spectrum is affected by measurement uncertainties, which will be estimated using the LF band. By using the RMC model, we model the spectra in the matrix, such as the Mayer wave and the RSA component; and we use the LF spectrum as a reference to identify relevant elements in the matrix. Then, these identified spectra, such as vectors **s**_2_, **s**_5_, and **s**_*m*_, along with **s**_3_ form a new matrix, where the HF band of **s**_3_ can be estimated using the RMC method described in the above iteration procedures.

### 3.4 Evaluation of Estimation Performance

The developed RMC method for HRV spectrum estimation is used to analyse a variety of datasets as discussed in the next section. To evaluate the model performance on spectrum estimation across different subjects and datasets, we use the normalised root mean square error (NRMSE) as the performance indicator. For the estimation of missing entries in the **s**_*j*_ spectrum, the NRMSE index can be calculated as follows [23],

(3.15) $$\text{NRMSE}=\sqrt{\frac{\frac{1}{N_{f}}\sum_{k=1}^{N_{f}}{\left({\hat{s}}_{j,k}-s_{j,k}\right)}^{2}}{\frac{1}{N_{f}-1}\sum_{k=1}^{N_{f}}{\left(s_{j,k}-{\bar{s}}_{j}\right)}^{2}}}$$

where, *s*_*j,k*_ ∈ **S** is the original spectrum, *j* = 1, 2, …, *n_r_* is the index of the spectrum, and *k* = 1, 2, …, *N_f_* indicates the data points of missing entries in the spectrum. *ŝ_j,k_* is the estimated spectrum, and ${\bar{s}}_{j}=\frac{1}{N_{f}}\sum_{k=1}^{N_{f}}s_{j,k}$ is the mean value of the spectrum. The calculation of this index normalises the root mean square error with the standard deviation of the spectrum, which enables to evaluate the model performance across different ECG datasets using an indicator with the same scale.

## 4 Data Analysis

### 4.1 Datasets

Generally, HRV data can be derived from ECG signals, and in the current study, we retrieved five widely used benchmark ECG datasets to evaluate the performance of our developed RMC model. These datasets are summarised as follows.

(i) Combined measurement of ECG, Breathing and Seismocardiograms (*CEBSDB*) [32]: this dataset consists of ECG recordings sampled from 20 healthy volunteers. After excluding a low-quality recording (‘m018’) due to the issue of electrode contact, the ECG signals collected from 19 subjects are used to for the study, which have a sampling duration of approximately 50 minutes.
(ii) Supraventricular arrhythmia database (*SADB*) [33]: this dataset includes 78 ECG recordings sampled from subjects with supraventricular arrhythmias, and each signal has a recording time duration of 30 minutes.
(iii) St Petersburg INCART 12-lead Arrhythmia Database (*SPIADB*) [34]: This dataset consists of 75 ECG recordings with a duration of 30 minutes, which are collected from patients undergoing tests for coronary artery disease.
(iv) European ST-T Database (*ESTDB*) [35]; This database is widely used for the evaluation of ST and T-wave changes in the ECG morphologies, and includes 90 annotated excerpts of ambulatory ECG recordings with two-hour sampling durations.
(v) Apnea-ECG Database (*APEDB*) [36]: this data consists of 70 ECG recordings with a set of apnea annotations, and each of the recordings has an approximate duration of 7 to 10 hours.

Algorithm 1 **Estimation of HRV spectrum using our developed RMC method.**

   **Input :**Matrix $S\in {\mathbb{R}}^{n_{r}\times n_{f_{r}}}$, 𝒜_Ω,**H**_, 𝒮_*m*_,

              tolerance *e*_*tol*_ and *λ*_*tol*_.

   **Output :**Estimated spectrum matrix **X**.

1 **Initialisation: s**_*j*_ ∈ **𝒮**_*m*_, *λ* ← 0, *λ*_*prev*_ ← 10^6^;

2 **for** *i* ← 1 **to** *n_r_* − 1 **do**

3       Compuate $s_{i}^{m}(f)$ by modelling *s_i_*(*f*) ∈ **S**;

4       Calcualte $\gamma \left(s_{i,\ell_{t}}^{m},s_{j,\ell_{t}}^{m}\right)$ between the spectra;

5 **end**

6 **Sort** $\gamma \left(s_{i,\ell_{t}}^{m},s_{j,\ell_{t}}^{m}\right)$ and obtain 𝒢_*K*_(**s**_*j*_);

7 Update$S\leftarrow S_{{𝒢}_{K}}$, and ${𝒜}_{\text{Ω},\text{H}}\leftarrow {𝒜}_{\tilde{\Omega },{𝒢}_{K}}$;

8 Assign **S** ← 𝒜_Ω,**H**_**S**, *λ*_min_ ← 0, *λ*_max_ ← ||**S**||*;

9 **while** ϵ(**X**) > *e*_*tol*_ *or* |*λ* − *λ*_*prev*_| > *λ*_*tol*_ **do**

10      Update *λ*_*prev*_ ← *λ*;

11      Compute and update *λ* ← (*λ*_min_ + *λ*_max_)/2;

12      Estimate and update **X** ← 𝒯 (**S**, *λ*);

13      Compute the estimation error *ϵ*(**X**);

14      **if** *ϵ*(**X**) *> e*_*tol*_ **then**

15           Update *λ*_min_ ← *λ*;

16      **else**

17           Update *λ*_max_ ← *λ*;

18      **end**

19 **end**

20 Output the estimated matrix **X**.

These datasets include ECG recordings with diverse characteristics, such as data sampled from healthy volunteers, patients with arrhythmias, coronary artery disease, ST and T-wave changes, and apnea. Therefore, the five ECG datasets will generate different types of HRV data, and in combination allow a comprehensive evaluation of our developed method for spectrum estimation. It is noted that the ECG signals in these datasets have varied lengths of sampling durations, and we use the first hour of the signal only in instances of a long-time sampling duration.

### 4.2 Signal Processing

**Figure 2:**
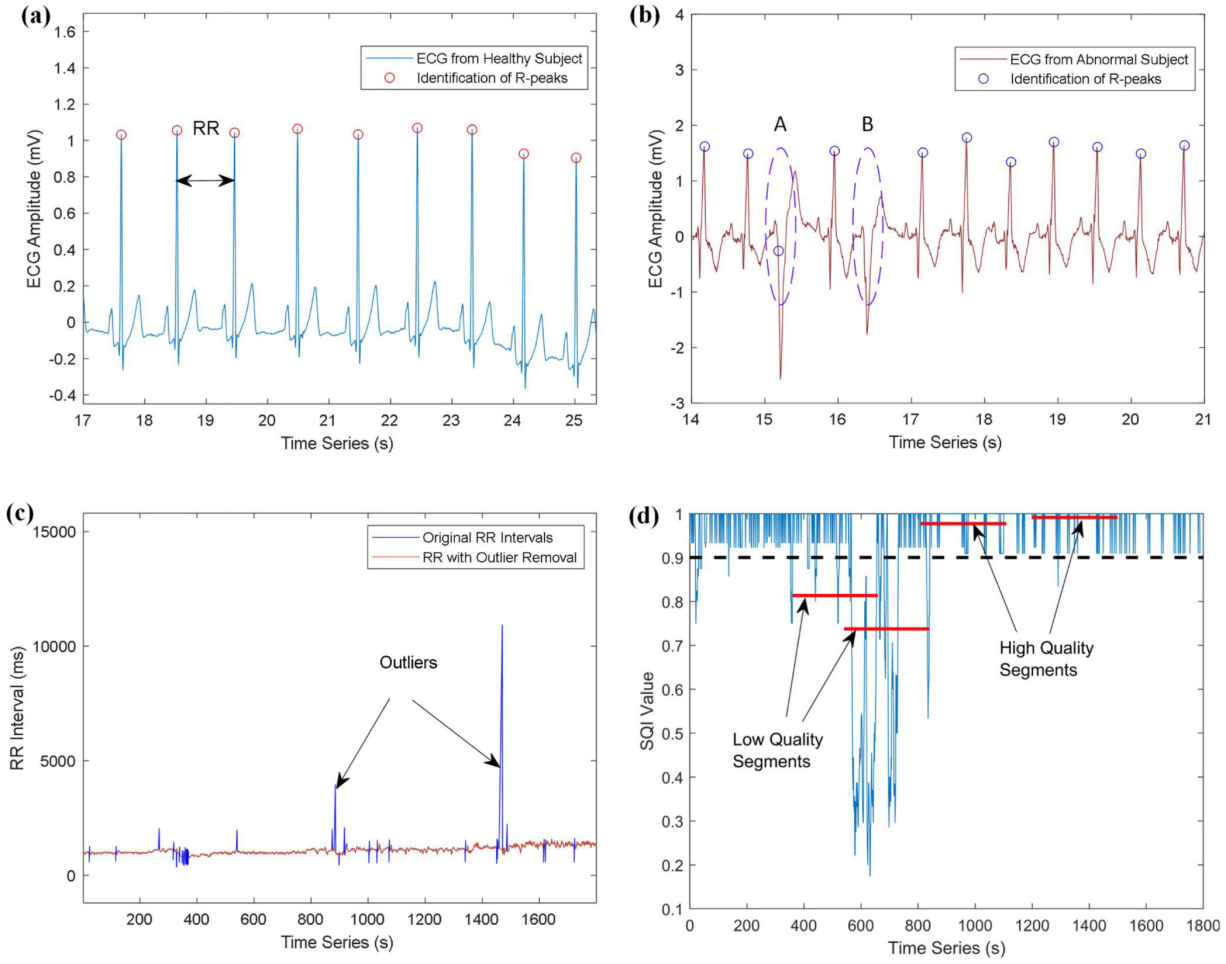
ECG signal processing and signal quality analysis of HRV data. (a) R-peaks identification for ECG recording sampled from healthy subject. (b) R-peaks identification for ECG recording sampled from abnormal subject. (c) Outlier detection in RR intervals. (d) Signal quality analysis for the HRV data.

To generate HRV data for this study, we first identify R-peaks from these ECG recordings by analysing the QRS complex. Figure 2(a) shows the identified R-peaks in the ECG recording using the QRS detector as developed in [37]. After identifying the R-peaks, a sequence of RR intervals can be calculated from timestamps of the consecutive peak values. Next, we implement outlier detection in the derived RR intervals using an open source benchmarked toolbox [38], where the outliers are defined as data points that are too close together, or large RR intervals caused by gaps, or artefact annotations of the signal. Figure 2(c) illustrates the detected outliers, which are processed by removal and interpolated to generate the preprocessed sequence.

We note that although a variety of techniques have been developed to detect the R-peaks [39, 40], it is still challenging to accurately identify the peak values for different types of ECG recordings. For example, as shown in Figure 2(a), the R-peaks are correctly identified for the ECG signal that is sampled from a healthy subject. However, as shown in Figure 2(b), the R peaks in waves *A* and *B* are wrongly identified for the ECG signal that is collected from an abnormal subject. The comparison of R-peak detection indicates that it may be not always reliable to use the technique to identify peak values for different types of ECGs.

To obtain high-quality HRV data, this study utilises two widely recognised detectors for identifying peak values: the ‘jqrs’ detector [37] and the ‘wqrs’ detector [40]. Subsequently, a Signal Quality Index (SQI) is computed for each detected R-peak by contrasting annotations from both detectors [41].

Following this, the average SQI value of R-peaks within 5-minute HRV data segments is calculated, serving as a quality indicator for each segment. For instance, a segment is deemed high-quality if its SQI indicator exceeds 0.9; otherwise, it is considered low-quality. As illustrated in 2(d), two data segments exhibit SQI values of 0.813 and 0.737, categorising them as low-quality. By calculating the average SQI values, the low-quality and high-quality data segments can be efficiently identified, and the low-quality segments will not be used for further analysis.

### 4.3 Calculation of the PSD Spectrum

**Figure 3:**
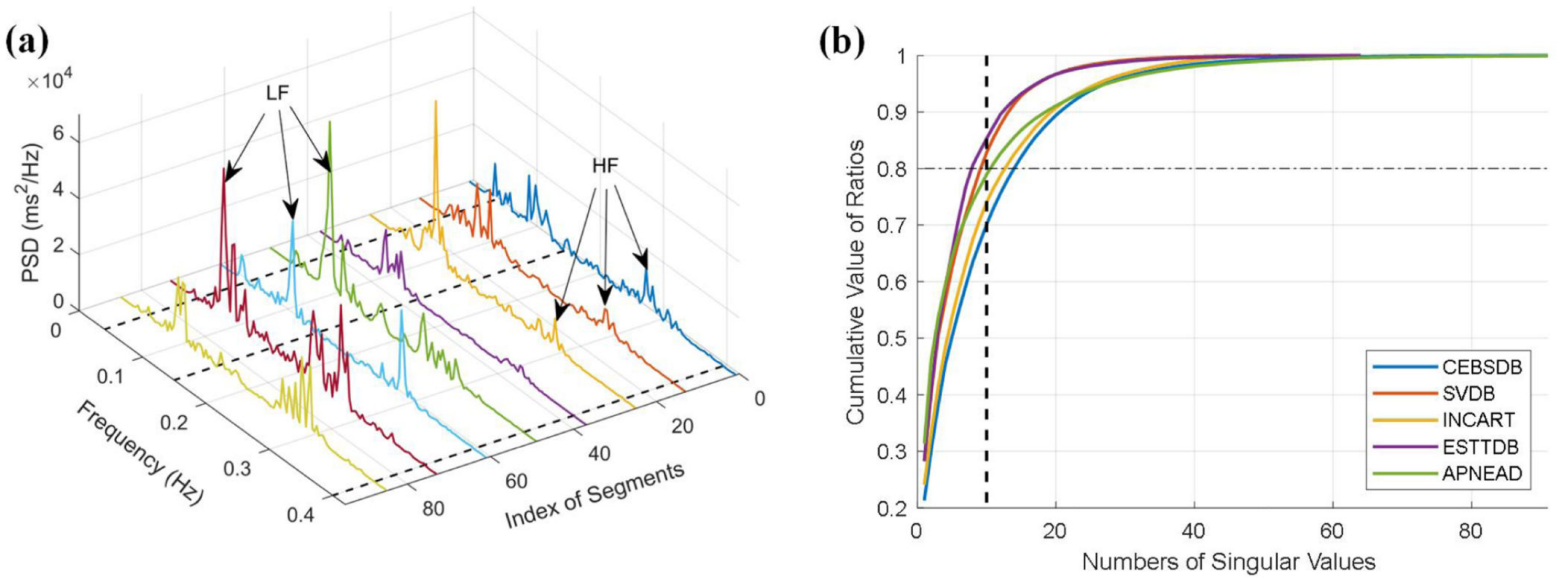
HRV spectrum and the cumulative ratios. (a) PSD spectra of different data segments. (b) Distributions of cumulative ratios between singular values of the nuclear norm.

The preprocessed HRV data is then resampled with a frequency of 4 Hz, and a 4^*th*^ order Butterworth filter is used to remove noises with the pass band of 0.03 Hz to 0.9 Hz [42]. The filtered data is used to derive HRV spectrum in the frequency domain, and Welch’s algorithm with an overlap of 50% is used for the calculation [42]. Next, the HRV spectrum matrix can be obtained by stacking all the calculated spectra. Figure 3(a) shows the three-dimensional plots of PSD spectra that are derived from different data segments. It can be seen from Figure 3(a) that the PSD spectra have similar patterns in the waveforms, i.e., LF and HF components, indicating the low-rank property of the HRV matrix derived from the PSD spectra.

In a further step, we calculate the singular values of the derived HRV matrix, and compute the ratio between singular values and the nuclear norm of the spectrum matrix, which is used as an indicator of the rank property of the matrix [23]. As shown in Figure 3(b), we demonstrate the low-rank property of the HRV data by calculating the cumulative ratios of singular values. It can be seen from the Figure 3(bthat the summation of the top ten singular values accounts for approximately 80% of the nuclear norm, indicating the low-rank properties of the HRV spectrum matrices that are derived from these five ECG datasets.

## 5 Results and Discussion

### 5.1 Uncertainty Estimation for PSD Spectrum

**Figure 4:**
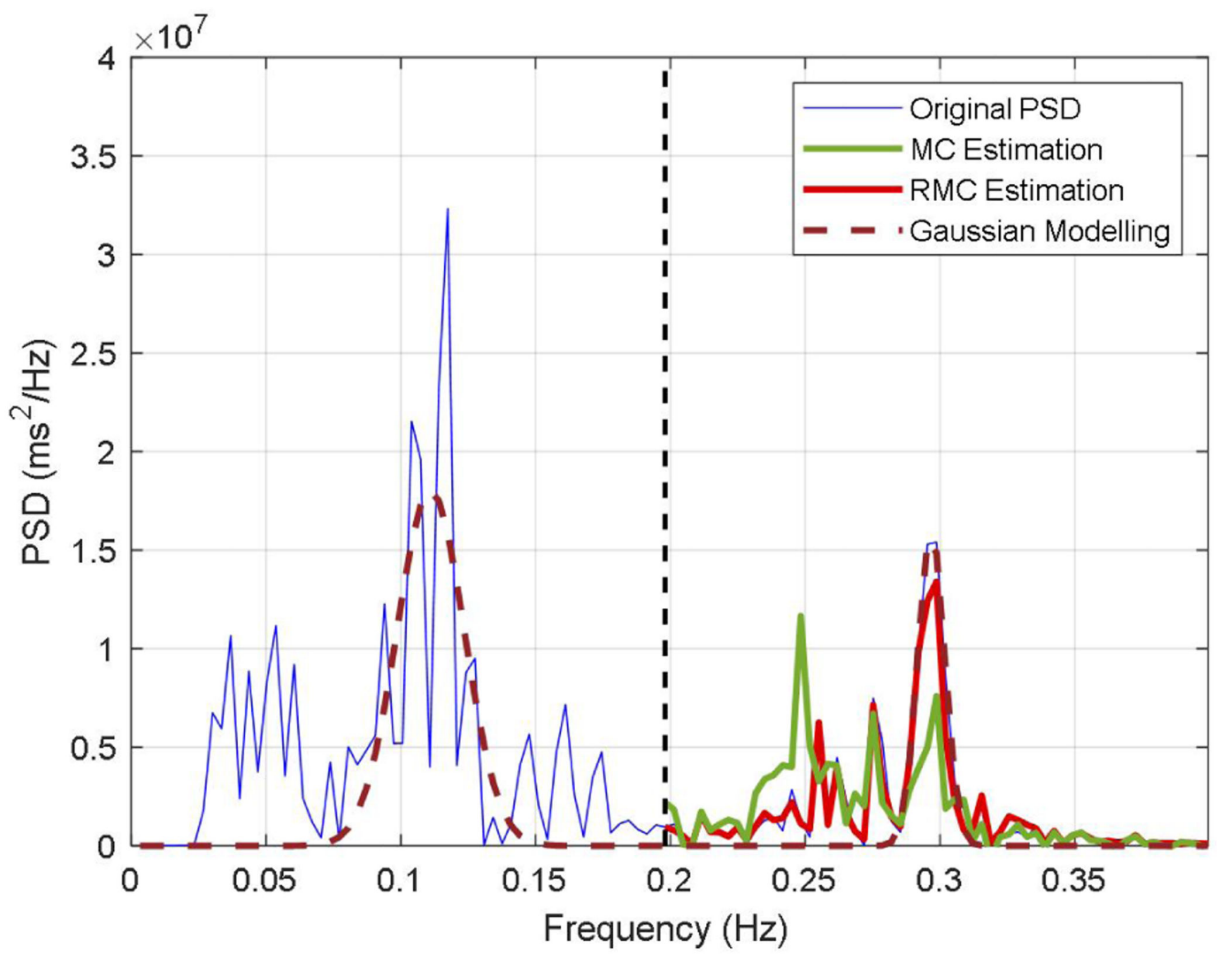
Comparison of HRV spectrum estimation using different methods.

The derived HRV matrix is then used to evaluate the performance of our developed model for uncertainty estimation. We note that the uncertainties or distortions of HRV spectrum can result from many different factors, such as noises, artefacts, missing values, and a variety of computational approaches (e.g., QRS detection, and data interpolation). Without loss of generality, we simulate the uncertainties as missing values in the HRV spectrum, and evaluate the performance of our developed method for the uncertainties estimation. As an example in Figure 4, we showed the estimation of uncertainty in HRV spectrum, which is represented as missing values and they are mostly in the HF of the spectrum. Then, we use the rest part of the spectrum (i.e., LF band) to estimate the HF band. It can be seen from Figure 4 that the estimated HF spectrum matches well with the original PSD spectrum, which efficiently estimated the waveform of the HF in the spectrum, but the estimation with the MC model introduced large variations around the frequency of 0.25 Hz.

We use the developed RMC method to estimate uncertainties in the spectrum. We simulate the spectrum with the model described in Eq. (3.10), and then use the modelled signal to identify relevant data segments in the HRV matrix. As an illustration in Figure 4, we show the signal modelling for the PSD spectrum of 31^*st*^ data segment of the matrix. It can be seen that the modelled signal efficiently represents the characteristics of the spectrum, such as the LF and HF.

**Figure 5:**
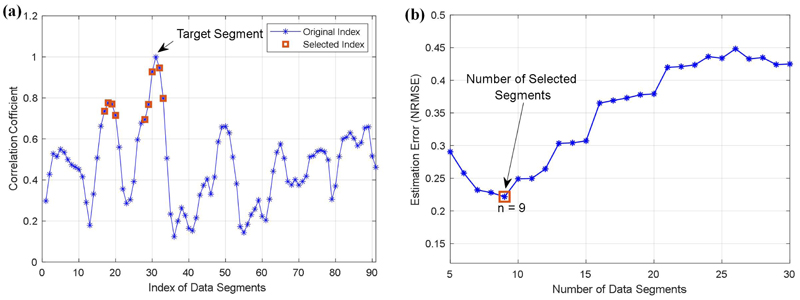
HRV spectrum estimation using matrix completion. (a) The correlation coefficients of different HRV segments. (b) Estimation errors for different combinations of data segments.

Next, we calculate correlation coefficients between different data segments using the modelled signals, and select the data with high coefficients to reconstruct the HRV matrix. Figure 5(a) shows the calculated coefficients for segments in the original data matrix, and Figure 5(b) illustrates estimation errors for different combinations of selected data segments. It can be seen from Figure 5(b) that the RMC model obtains the least estimation error of 0.222 when using nine data segments, and it is smaller than the estimation error of 0.830 using the traditional MC method. As shown in Figure 5(a), we highlight the selected data segments with square markers. It can be seen from Figure 5(a) that the selected important data elements include five neighbours of the target data segment, this is consistent with the rational approach that missing values can be generally imputed using nearest neighbours. We also note that the model identifies four segments as important data, which are not the neighbours of the target segment, indicating that some data with far distances in the sequence may also provide valuable information for the estimation of missing entries.

**Figure 6:**
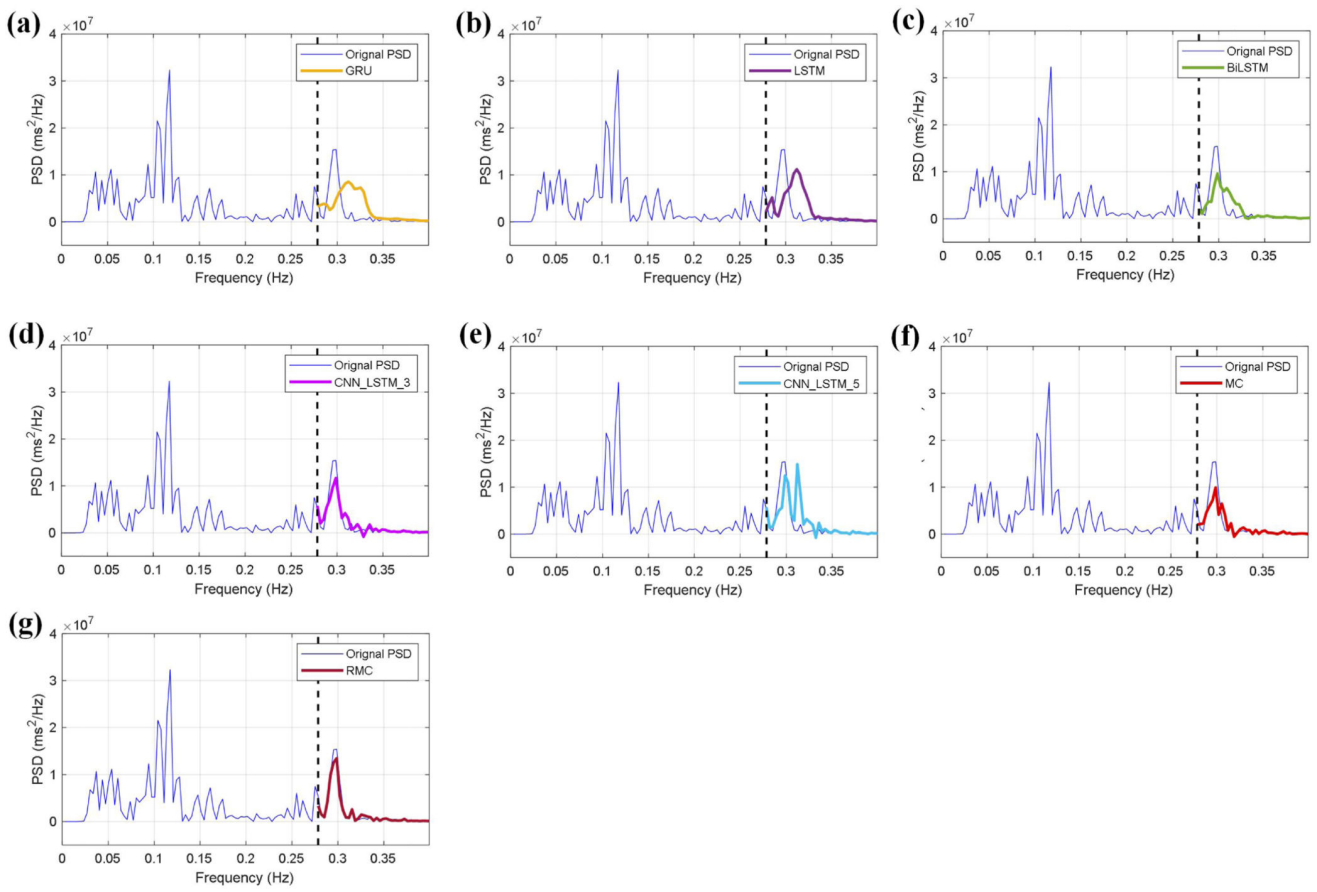
Uncertainty estimation of HRV spectrum using different models with the masking ratio of 30%. (a) The GRU model, (b) the LSTM model, (c) the BiLSTM model, (d) the CNN_LSTM_3 model, (e) the CNN_LSTM_5 model, (f) the MC method, and (g) the RMC method.

**Figure 7:**
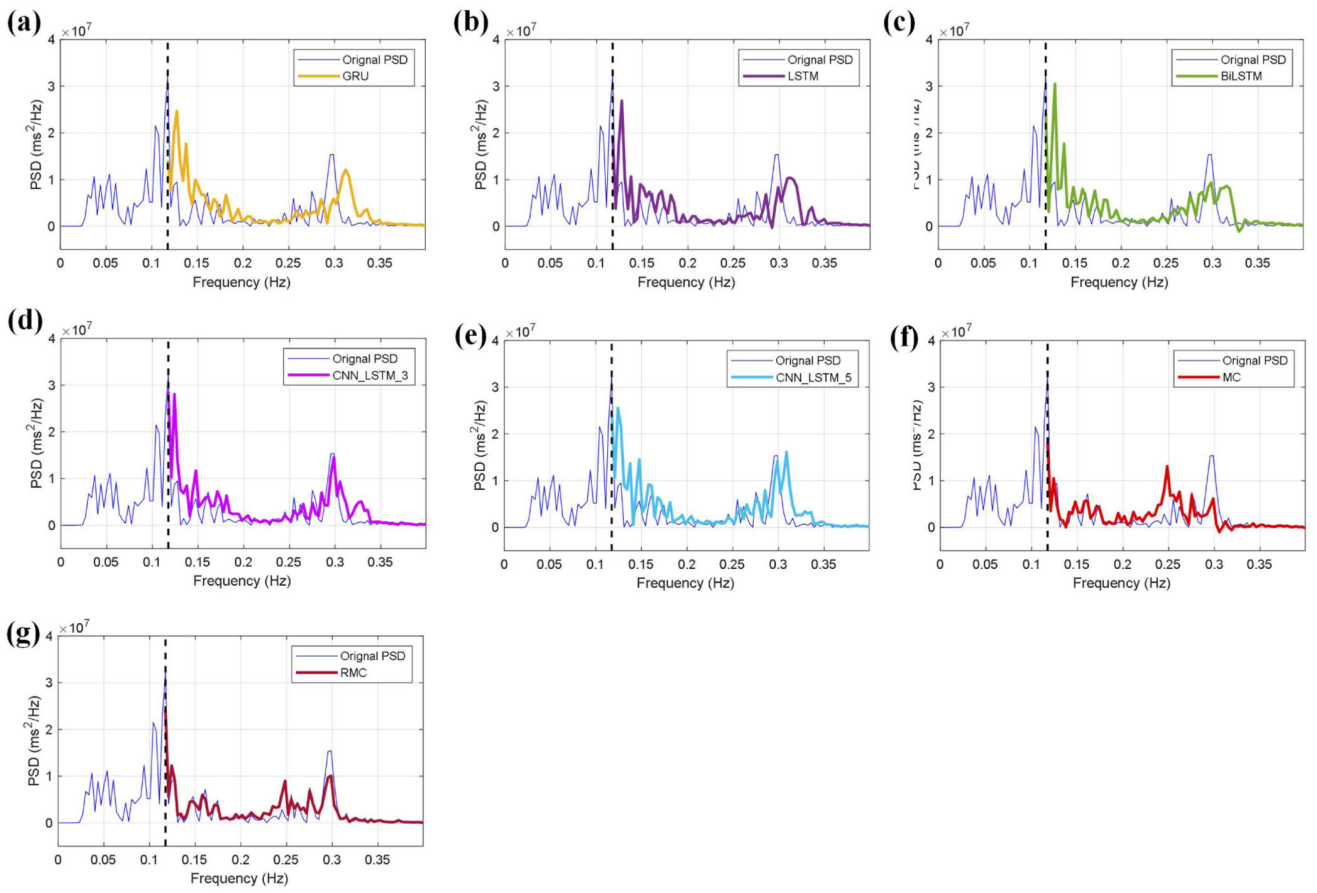
Uncertainty estimation of HRV spectrum using different models with the masking ratio of 70%. (a) The GRU model, (b) the LSTM model, (c) the BiLSTM model, (d) the CNN_LSTM 3 model, (e) the CNN_LSTM_5 model, (f) the MC method, and (g) the RMC method.

In a further step, we evaluate the model performance on uncertainty estimation in different conditions, and we apply different masking ratios on the HRV spectrum. As shown in Figures 6 and 7, our developed RMC method efficiently estimates missing values for the PSD spectra with masking ratios of 30% (Figure 6(g)), and 70% (Figure 7(g)), which have estimation errors of 0.221 and 0.426 separately. The two experiments show the robustness of our developed RMC method for uncertainty estimation in the HRV spectrum.

### 5.2 Comparison Methods and Results

The current study focused on estimating uncertainties of the HRV spectrum, and we compared our developed methods with different types of regression machine learning models; In particular, the deep recurrent neural networks have been demonstrated with excellent performance on prediction or regression analysis. To be specific, we used the gated recurrent units (GRU), the long short-term memory (LSTM) model, and the bidirectional LSTM (BiLSTM) for the comparison study. We note that convolutional neural networks (CNN) have shown efficiency in extracting features from data sequences [43]. Therefore, other than the GRU, LSTM, and BiLSTM, we used two hybrid models for the comparison study.

For the three types of recurrent neural networks, i.e., the GRU, LSTM, and BiLSTM, they have 120 hidden units for the regression analysis [44]. For the two types of hybrid models, the hyperparameters are tuned with trial-and-error search; the first one (CNN LSTM 3) uses three 1D-CNN layers with 32, 64, and 64 filters respectively, each filter has a size of 3 [45]. Each convolutional layer is followed by a rectified linear unit (ReLU) function, and a bath normalisation layer, which is then flattened and connected with a LSTM layer for the regression analysis. The second type of hybrid models (CNN LSTM 5) consists of five CNN layers, which have 32, 32, 64, 64, and 64 filters respectively, which also uses a LSTM layer for the regression analysis. The deep learning models are trained with

Adam optimizer with an initial learning rate of 0.005, the maximum epoch size is 100, and the batch size is set as 20. For the prediction analysis, we use data points from previous steps, i.e., 10 steps [46], to predict the current value in the data sequence.

The estimation results of these regression models are demonstrated in Figures 6 and 7, which correspond to the masking ratios of 30% and 70% respectively. As a comparison study, we also include the estimation results from the traditional MC method. It can be seen from Figures 6 and 7 that these machine learning models have small errors on the spectrum estimation; In particular, the BiLSTM and hybrid models efficiently estimate the peak values around 0.3 Hz for the two masking ratios. Compared with the deep learning models and the MC method, our developed RMC method has more efficient estimation performance, which obtains the least estimation errors of 0.221 and 0.426 for the 30% and 70% masking ratios respectively.

### 5.3 Statistical Results

We implement the estimation of missing entries for all ECG recordings in the five benchmark datasets using our developed RMC method and the regression models. Tables 1-3 show the mean value and standard deviation of estimation errors for the five benchmark datasets with 30%, 50%, and 70% masking ratios. The minimum estimation error for each dataset is bold-faced. It can be seen from Tables 1-3 that compared with the five machine learning models and the traditional MC method, our developed RMC method obtains the least estimation error for all of the five ECG datasets, and the increased masks ratios occurred alongside an increase in the estimation errors, which have the values of 0.286 ± 0.194, 0.312 ± 0.203, and 0.379 ± 0.228 for the CEBSDB with the three masking ratios.

**Figure 8:**
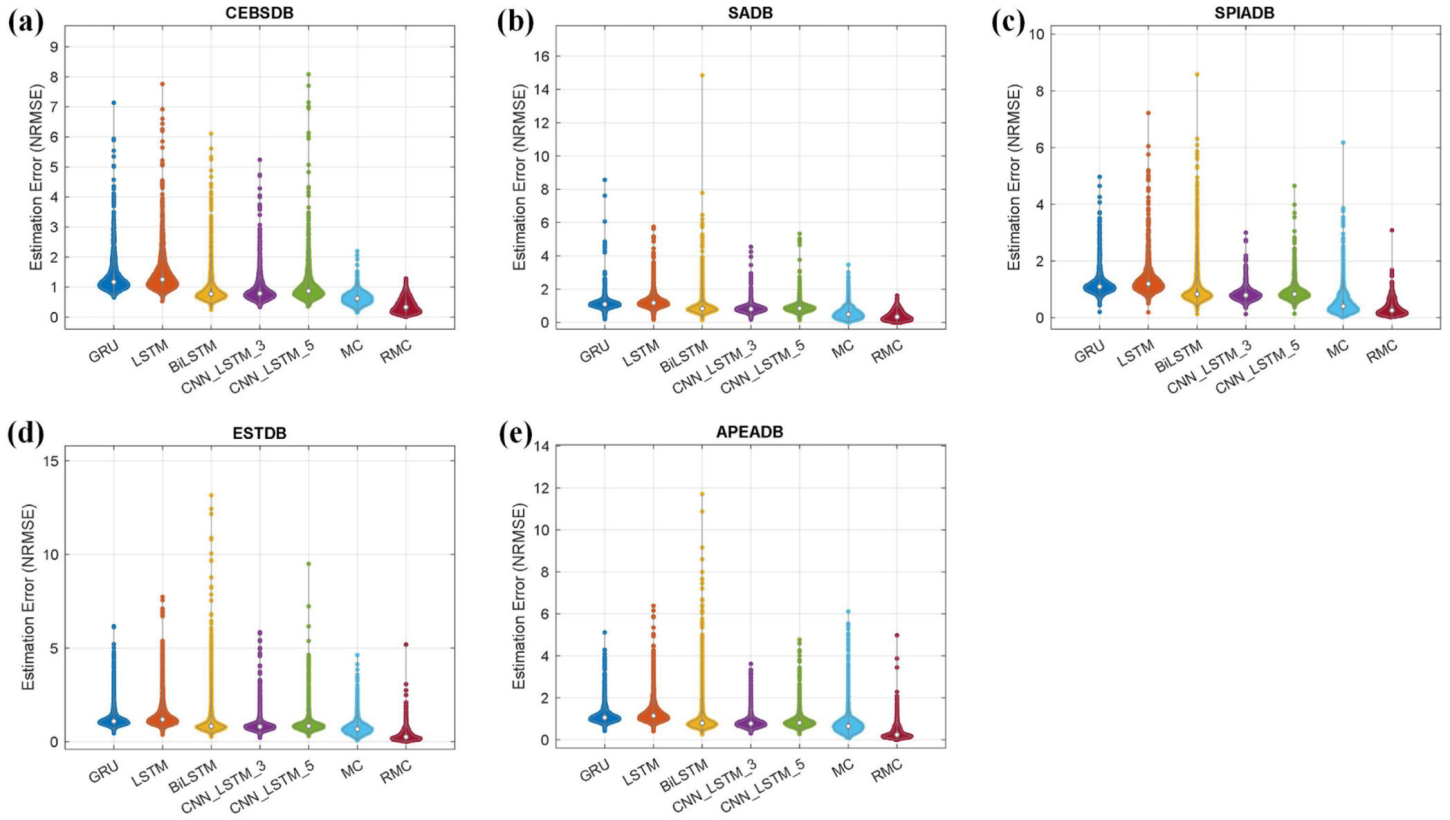
Distributions of estimation errors of different methods on five ECG benchmark datasets, (a) CEBSDB, (b) SADB, (c) SPIADB, (d) ESTDB, and (e) APEADB.

Additionally, we provide violin plots as shown in Figure 8 to demonstrate the distributions of estimation errors for the seven models. It can be seen from Figure 8 that our developed RMC method obtains the least estimation errors with median values of 0.330, 0.337, 0.260, 0.259, and 0.235 for the five benchmark datasets respectively. In particular, we note that compared with the five machine learning models and the traditional MC method, the RMC method has much smaller variations of the estimation errors for all the ECG datasets, which indicate the stability and robustness of our developed RMC method for the spectrum estimation.

### 5.4 Comparison on Computational Costs

We calculate the computation cost for missing entry estimation using the RMC method and the other six methods. All of the estimation tasks are implemented with Matlab R2022a, Intel(R) Core(TM) i7-1165G7@2.8GHz, and 32GB RAM. We calculate the mean value and standard deviation of computation time for all HRV data segments in the benchmark dataset. Table 4 presents the comparison of computation cost for all the estimation methods. We note that the five deep learning models have an increasing trend of computation cost with the increasing model complexity, which can be seen from Table 4 that among these deep learning models, the GRU model has the least computation time with 1.117 ± 0.460 s. Compared with the deep learning models and the MC method, our developed RMC method has the least computation time with 0.105 ± 0.096 s. The results demonstrate the efficiency of our developed RMC method for HRV spectrum estimation.

### 5.5 Discussion

Spectrum metrics of HRV data are important indicators for monitoring physiological and pathological conditions. However, HRV data are sensitive to a variety of uncertainties in the measurements, such as motion artefacts and missing values. Although numerous methods have been developed to address uncertainties of HRV analysis, it is difficult to remove artefacts without affecting the signal waveforms. We propose to estimate uncertainties of HRV data from a new view in the spectrum analysis; In particular, we consider the characteristics of LF and HF bands of HRV spectrum. With extensive experimental studies, we show that the PSD data demonstrate similarities between spectrum waveforms, indicating the low-rank property of the spectrum matrix, which enables us to use the MC technique for the uncertainty estimation.

A variety of computational techniques have been developed for missing value estimation. For example, different types of interpolation methods [47] and machine learning models [48], including linear interpolation, k-nearest neighbour (kNN), multiple imputation by chained equation (MICE), neural networks, and random forest. However, we note that deep neural networks (DNNs) particularly with recent advancements have shown superior performance than these traditional methods in estimating sequential data [49]. It is therefore we compare our proposed RMC method with different types of DNNs. We also acknowledge more advanced predictive models that have been developed recently, such as Transformer [50], Informer [51], TimesNet [52], etc., which have not been used for HRV uncertainty estimation, motivating us to implement more comprehensive comparison in our future study.

It is a challenging task of estimating missing entries for sparse matrices. We note the performance of low-rank MC method in handling sparse matrices, which has been demonstrated in previous literature [53, 54, 55]. In this study, we proposed a refined version of the traditional MC method, and our developed RMC method was used for HRV uncertainty estimation. The results indicated that compared with different types of DNN models, our RMC method demonstrated promising performance across all benchmark datasets. The improved model performance can be attributed to the incorporation of specific characteristics of the HRV spectrum into our modelling approach. For instance, the LF of the HRV spectrum exhibits a peaked Mayer waveform, while the HF displays a dominant RSA waveform. These peaked waveforms, observed within a limited number of data points, can pose challenges for DNN models in uncertainty estimation, and therefore lead to reduced performance.

There are many different types of uncertainties in the analysis of HRV data, such as measurement noises and artefacts in data acquisition, and computational uncertainties in data processing (e.g., data interpolation, and data resampling). Without loss of generality, we simulate the uncertainties of HRV spectrum as missing values, which can also be used to indicate the distorted entries affected by motion artefacts or data interpolation. We show the advantages and robustness of our developed RMC method for uncertainty estimation on a range of benchmark datasets. To perform a fair comparison, the NRMSE indicator was used to evaluate the performance of different models on five ECG datasets, we will explore other metrics for evaluating different types of uncertainties in our future studies. In addition, we note that HRV spectrum is derived from NN intervals of ECG signals. However, NN interval outliers, caused by missed or false beat detections could impact the HRV spectrum. Our next step research aims to thoroughly investigate these uncertainties and develop robust MC with other advanced techniques, such as *𝓁*_*p*_ minimisation [27], and adaptive weighting strategy [56] to mitigate the effects of outliers.

## 6 Conclusions

This paper presents a new approach to address the challenges of uncertainty estimation in the HRV spectrum using the low-rank MC method. In particular, we propose a new RMC method that takes into account the characteristics of frequency components in the HRV spectrum for more accurate uncertainty modelling. We evaluate the performance of our developed RMC method on five benchmark datasets with varying masking ratios. To assess its effectiveness, we compare our method with five deep regression models and the traditional MC method. The results demonstrate that our RMC method achieves the lowest estimation error while maintaining the minimal computational cost, which highlights the advantages and efficiency of our developed model for HRV spectrum estimation.

## Figures and Tables

**Table 1:** Comparison of estimation errors for different methods on benchmark datasets with 30% masking ratio.

Datasets	GRU	LSTM	BiLSTM	CNN_LSTM_3	CNN_LSTM_5	MC	RMC
CEBSDB	1.379 ± 1.112	1.346 ± 0.970	0.671 ± 0.355	0.672 ± 0.459	0.770 ± 0.692	0.393 ± 0.291	0.286 ± 0.194
SADB	1.389 ± 1.751	1.318 ± 1.280	0.733 ± 0.901	0.746 ± 0.902	0.902 ± 1.808	0.332 ± 0.253	0.304 ± 0.199
SPIADB	1.241 ± 0.776	1.211 ± 0.609	0.647 ± 0.289	0.655 ± 0.375	0.709 ± 0.378	0.361 ± 0.539	0.305 ± 0.327
ESTDB	1.317 ± 1.096	1.297 ± 1.043	0.687 ± 0.430	0.691 ± 0.456	0.753 ± 0.549	0.494 ± 0.464	0.329 ± 0.251
APEADB	1.283 ± 0.740	1.268 ± 0.646	0.644 ± 0.263	0.663 ± 0.337	0.727 ± 0.392	0.426 ± 0.357	0.290 ± 0.228

**Table 2:** Comparison of estimation errors for different methods on benchmark datasets with 50% masking ratio.

Datasets	GRU	LSTM	BiLSTM	CNN_LSTM_3	CNN_LSTM_5	MC	RMC
CEBSDB	1.061 ± 0.234	1.091 ± 0.275	0.621 ± 0.163	0.619 ± 0.164	0.663 ± 0.175	0.432 ± 0.192	0.312 ± 0.203
SADB	1.067 ± 0.149	1.093 ± 0.190	0.670 ± 0.153	0.673 ± 0.129	0.714 ± 0.136	0.386 ± 0.254	0.338 ± 0.226
SPIADB	1.076 ± 0.168	1.097 ± 0.201	0.657 ± 0.150	0.662 ± 0.115	0.706 ± 0.128	0.388 ± 0.529	0.310 ± 0.290
ESTDB	1.113 ± 0.707	1.138 ± 0.692	0.680 ± 0.306	0.704 ± 0.427	0.745 ± 0.443	0.590 ± 0.446	0.336 ± 0.276
APEADB	1.059 ± 0.292	1.085 ± 0.334	0.642 ± 0.216	0.648 ± 0.168	0.687 ± 0.175	0.547 ± 0.422	0.312 ± 0.256

**Table 3:** Comparison of estimation errors for different methods on benchmark datasets with 70% masking ratio.

Datasets	GRU	LSTM	BiLSTM	CNN_LSTM_3	CNN_LSTM_5	MC	RMC
CEBSDB	1.350 ± 0.591	1.446 ± 0.676	0.926 ± 0.563	0.906 ± 0.451	1.030 ± 0.622	0.633 ± 0.202	0.379 ± 0.228
SADB	1.131 ± 0.361	1.247 ± 0.422	0.983 ± 0.652	0.819 ± 0.238	0.873 ± 0.284	0.539 ± 0.318	0.397 ± 0.262
SPIADB	1.179 ± 0.398	1.325 ± 0.544	1.019 ± 0.674	0.828 ± 0.230	0.890 ± 0.308	0.502 ± 0.406	0.338 ± 0.247
ESTDB	1.193 ± 0.418	1.335 ± 0.543	1.028 ± 0.743	0.849 ± 0.304	0.903 ± 0.369	0.728 ± 0.356	0.337 ± 0.252
APEADB	1.144 ± 0.368	1.266 ± 0.470	0.958 ± 0.689	0.811 ± 0.256	0.864 ± 0.315	0.694 ± 0.409	0.315 ± 0.246

**Table 4:** Comparison of computation time for different estimation methods.

Methods	Computation Cost (s)
GRU	1.117 ± 0.460
LSTM	1.357 ± 0.704
BiLSTM	2.298 ± 2.091
CNN_LSTM_3	2.484 ± 2.218
CNN_LSTM_5	2.889 ± 0.909
MC	0.669 ± 0.340
RMC	0.105 ± 0.096
